# Management of people with low back pain: a survey of opinions and beliefs of Dutch and Belgian chiropractors

**DOI:** 10.1186/s12998-022-00437-1

**Published:** 2022-06-20

**Authors:** Lobke P. De la Ruelle, Annemarie de Zoete, Michiel R. de Boer, Maurits W. van Tulder, Raymond Ostelo, Sidney M. Rubinstein

**Affiliations:** 1 Department of Health Sciences, Faculty of Science and Amsterdam Movement Science Research Institute, Vrije Universiteit, Boechorststraat 7, Room MF-J284, 1081 BT Amsterdam, The Netherlands; 2 Department of General Practice and Elderly Care Medicine, UMCG, Groningen, The Netherlands; 3 Department Human Movement Sciences, Faculty Behavioural and Movement Sciences, Vrije Universiteit, Amsterdam, The Netherlands; 4 Department Physiotherapy and Occupational Therapy, Aarhus University Hospital, Aarhus, Denmark; 5 Department of Epidemiology and Data Science, Amsterdam Movement Sciences, Amsterdam UMC, Location VUmc, Amsterdam, The Netherlands

**Keywords:** Low back pain, Chiropractic, Guideline adherence

## Abstract

**Background:**

Chiropractors commonly provide care to people with low-back pain (LBP). The aim of this survey was to determine the opinions and beliefs of chiropractors regarding the support and management of LBP. We also investigated whether their management is in accordance with the three most commonly recommended approaches to LBP based upon international guidelines (i.e. advice regarding return-to-work, limit bedrest, and stay active).

**Methods:**

A web-based survey was sent out in 2013 to collect data from registered Dutch and Belgian chiropractors. In addition to providing a description of their sociodemographic and practice characteristics, chiropractors were asked to complete six patient vignettes representing people with LBP who typically present to a chiropractor. The respondents indicated which intervention(s) they would recommend or undertake. Based upon these vignettes, we were able to determine whether their management approach adhered to clinical guidelines. Generalized mixed models were used to explore guidelines adherence and their relationship to chiropractors’ characteristics.

**Results:**

In total, 60% (n = 203/340) of the chiropractors who were invited, chose to participate. Chiropractors reported applying a chiropractic adjustment in 90% of all vignettes, while the advice to exercise varied from one-third in the chronic cases to approximately half of those with acute LBP. More than 75% of the chiropractors would initially treat LBP 1–2 times a week. More than 90% of the chiropractors advised against bedrest. Overall, self-reported adherence to clinical guidelines for all six vignettes was [64.5% (CI 58.7–70.0)]. Adherence in the chronic vignettes [73.4% (CI 66.7–79.2)] was better than in the acute vignettes [55.9% (CI 50.5–61.1)]. Importantly, regarding recommended approaches to LBP, chiropractors more consistently followed guidelines regarding advice to limit bedrest [98.5% (CI 97.3–99.1)] than advice to stay active [77.5% (CI 72.3–81.9)] or return-to-work [59.4% (CI 55.2–63.4)]. Finally, Dutch chiropractors were more likely to adhere to the guidelines than Belgian chiropractors.

**Conclusions:**

Chiropractic adjustments were the most common self-reported treatment modalities supplemented by exercise in the management of LBP patients. Two-thirds of the chiropractors reported adhering to the guidelines regarding management and advice for LBP patients. Practitioners should improve guideline adherence, particularly for acute LBP cases, and when advising on return-to-work.

**Supplementary Information:**

The online version contains supplementary material available at 10.1186/s12998-022-00437-1.

## Introduction

In the Netherlands and Belgium, low-back pain (LBP) is common and costly [1]. One of the professions treating LBP is chiropractic, a legalized and well-established profession within the healthcare system in many countries, such as Denmark, the USA, and Australia. As a result, chiropractors provide a significant proportion of the care for people with low back pain (LBP) in these countries [2, 3]. Despite the fact that chiropractic is a relatively small profession, the number of chiropractors in the Netherlands has increased from 150 to 299 over the past 20 years, and in Belgium from 97 to 130 chiropractors over the last 5 years. In the Netherlands, with its 17 million inhabitants, more than 1 million chiropractic treatments were delivered in 2019. The great majority of these consultations were for LBP [4], the same would apply for Belgium [5].

Many national and international clinical guidelines for the management of LBP [6–11] have been developed. The chiropractic guidelines do not differ from the multidisciplinary guidelines. These clinical practice guidelines can support health care providers in deciding on the appropriate care for the patient. By adhering to clinical guidelines, management of LBP will be more effective and safe [12, 13]. In the Netherlands and Belgium, there are multidisciplinary guidelines for LBP [7, 9]. While the Netherlands Chiropractic Association (NCA) has developed guidelines for acute and chronic LBP, these have not yet been published, while these are currently lacking for the Union of Belgian Chiropractors (BVC). Two international monodisciplinary guidelines have been published for chiropractors for acute and chronic LBP: the Mercy guidelines and synthesis of Council on Chiropractic Guideline and Practice Parameters [10, 14]. These clinical guidelines advise clinicians on treatment modalities to be used and on what advice should be given to LBP patients. The three most frequently addressed in the guidelines are advice to: (1) return-to-work; (2) limit bed rest and (3) stay active.

Studies suggest that chiropractors employ a wide variety of techniques and have varying views on clinical practice [15–21]. Only two studies have been conducted which investigated Dutch and Belgian chiropractors treatment approaches; however, these studies did not examine which treatment modalities were most commonly used [5, 22]. Several studies suggest that healthcare providers do not always treat or provide advice which is consistent with international guidelines [17, 19]; however, it is not clear how this may apply to chiropractors in the Netherlands or Belgium. For example, three studies suggest that characteristics of healthcare providers, such as familiarity with the guidelines, and beliefs and perceptions may be important factors that influence adherence to the guidelines [23–25]. Understanding these characteristics may help to position the chiropractic profession in these countries, and improve the awareness and implementation of guidelines by chiropractors.

Therefore, the aims of this study are: (1) To determine how chiropractors in the Netherlands and Belgium manage their patients; (2) To estimate whether their management approach is in line with the recommendations of clinical practice guidelines concerning return-to-work, limit bed rest, and stay active; and (3) To evaluate which factors are associated with the recommendations of the international chiropractic and multidisciplinary guidelines.

## Methods

### Design and setting

Data were collected via a web-based cross-sectional survey (Survey Monkey™). All chiropractors in the Netherlands, who were registered with the SCN (Stichting Chiropractie Nederland; Foundation for Chiropractors in the Netherlands) and member of the Dutch Chiropractor Association (NCA) and all Belgian chiropractors registered with the Union of Belgian Chiropractors (BVC), were invited to participate in 2013. At the time of data collection, NCA had 245 members practicing in the Netherlands, and the BVC had 111. If a chiropractor worked in both countries, he/she was analyzed as a chiropractor working in the Netherlands.

A link to this web-based survey was sent to all participants. A reminder email was sent 3 weeks later if the invited participants had not yet responded, and a telephone call was made to those chiropractors who had not yet completed the survey after 6 weeks.

### Survey

Prior to data collection, the survey was pre-tested in a pilot study with three Dutch chiropractors, which led to only minor textual changes. The survey (Additional file 1: Survey) explored various aspects of the management of LBP patients in chiropractic practice and took the chiropractors approximately 40 min to complete. To limit missing data, participants could only proceed if the previous question had been answered.

### Sociodemographics, practice information, and familiarity with clinical guidelines

This section included questions about demographics (e.g., age, gender, nationality), general characteristics (e.g., years in practice, postgraduate training and the type of practice), familiarity with clinical guidelines (yes/no), and whether the chiropractors familiar with the guidelines, adhered to these guidelines when managing the patients with LBP (yes/no).

### Self-reported management of patients with LBP

We used six patient vignettes reflecting three patients with acute LBP and three with chronic LBP whom chiropractors would typically see in their practices. Vignettes 1, 2 and 3 were acute, the other three were chronic. Vignettes 1 and 4 were uncomplicated LBP cases, without radiation to the legs or previous trauma. Vignettes 2 and 5 included patients who are already being treated but did not respond to treatment so far, and vignettes 3 and 6 included radicular symptoms. These vignettes were based upon previous studies [17, 26] and were modified for the Netherlands and Belgium.

For each vignette, the chiropractors were asked how they would manage the patient. The treatment options included (1) no intervention, (2) chiropractic adjustment (including SMT, Cox, Activator, Gonstead, and Thompson drop), (3) exercise, (4) education, (5) spinal traction, (6) psychosocial evaluation, and (7) non-exercise modalities. In addition, questions regarding advice to return-to-work, avoiding bed rest, and staying active were included, as these are among the most common recommendations in clinical guidelines for low back pain [27, 28]. Respondents were able to tick as many boxes as they felt were appropriate.

*Adherence to guidelines*. The appropriateness of responses was defined a priori by the project group using recommendations of the international chiropractic and multidisciplinary guidelines [6, 7, 10, 29]. Five chiropractors from the United States, Belgium, and Australia working in clinical practice, with multiple years of experience in chiropractic research and not participating in the survey, were asked to review our classification of the responses. After minor revisions, a consensus was reached on the classification.

The responses to the vignettes were classified as being ‘strictly in line with guideline recommendations’, ‘broadly in line with guideline recommendations’, or ‘not in line with guideline recommendations’, which are outlined in Table 1.

**Table 1:** Classification for treatment and advice on work, activity and bed rest offered at this visit described in the vignette

Question	Vignette	Response option on questionnaire	Authors classification of response
Treatment offered at this visit	Vignette 1 and 3	No intervention or chiropractic adjustment	Strictly in line with guideline recommendations
		No intervention or chiropractic adjustment + one other treatment option	Broadly in line with the guideline recommendations
		2 treatment options other than no intervention or chiropractic adjustment	Not in line with the guideline recommendations
	Vignette 2	No intervention or chiropractic adjustment and/or exercise	Strictly in line with guideline recommendations
		No intervention or chiropractic adjustment and/or exercise + one other treatment option	Broadly in line with the guideline recommendations
		2 treatment options other than no intervention chiropractic adjustment and/or exercise	Not in line with the guideline recommendations
	Vignette 4, 5 and 6	No intervention or chiropractic adjustment, exercise and/or psychosocial evaluation	Strictly in line with guideline recommendations
		No intervention or chiropractic adjustment, exercise and/or psychosocial evaluation + one other treatment option	Broadly in line with the guideline recommendations
		2 treatment options other than no intervention or chiropractic adjustment, exercise and/or psychosocial evaluation	Not in line with the guideline recommendations
Advice to return to work	All vignettes	Return to normal work	Strictly in line with guideline recommendations
		Return to part time or light duties	Broadly in line with guideline recommendations
		Be off work for a further...weeks (stating number of weeks)	Not in line with guideline recommendations
		Be off work until pain has improved	Not in line with guideline recommendations
		Be off work until pain has completely disappeared	Not in line with guideline recommendations
Advice to bed rest	All vignettes	Avoid resting in bed entirely	Strictly in line with guideline recommendations
		Avoid resting in bed as much as possible	Broadly in line with guideline recommendations
		Rest in bed only when pain is severe	
		Rest in bed until pain improves substantially	Not in line with guideline recommendations
		Rest in bed until pain disappears	Not in line with guideline recommendations
Advice to stay active	All vignettes	Perform usual activities	Strictly in line with guideline recommendations
		Perform activities within the patient’s tolerance	Broadly in line with guideline recommendations
		Perform only pain free activities	Not in line with guideline recommendations
		Limit all physical activities until pain disappears	Not in line with guideline recommendations

For the treatment, the vignettes of acute patients that were not already being treated (1 and 3) were classified as in line with the guidelines when the answer was either ‘no intervention’ or ‘chiropractic adjustment’. If one other treatment option was given besides chiropractic adjustment, it was classified as broadly in line with the guidelines. If the answer included more treatment options, it was considered not in line with the guideline recommendations.

In the vignette with the acute patient already being treated but not responding (2) the treatment option ‘exercise’ was also an answer that would classify as ‘in line with the guidelines’. Again, one extra treatment option was broadly in line with the guidelines, and two or more other treatment options were classified as not in line with the guidelines.

In the chronic vignettes (4, 5 and 6) ‘no intervention’, ‘chiropractic adjustment’, ‘exercise’, and/or ‘psychosocial evaluation’ were considered in line with the guidelines. One other treatment option was classified as broadly in line with the guidelines and two or more extra treatment options were classified as not in line with the guidelines.

To achieve dichotomization of the data, the categories ‘broadly in line’ and ‘in line’ were both considered ‘in line’.

### Analysis of the data

#### Demographic and clinical guidelines data

Chiropractors’ characteristics and choice of interventions or advice are described using means (SDs) for continuous data and percentages for categorical data.

#### Familiarity with the guidelines

We described familiarity with the guidelines in percentages. We used multivariable logistic regression analyses to assess whether there were associations between participant characteristics and familiarity with the practice guidelines. All independent variables were entered simultaneously. The odds ratios (OR) and 95% CIs are presented. The ORs describe the likelihood of familiarity with the guidelines, based on individual characteristics, such as years in practice, type of practice, country of origin, and post-graduate education.

#### Clinical vignettes

First, we used a binary logistic mixed model to assess the overall percentage of adherence to the practice guidelines by chiropractors and included a random intercept for chiropractors in the model. This method allowed for the correlation of responses within each individual chiropractor. Second, we ran the same model for assessing percentage adherence for the vignettes describing acute and chronic LBP patients, separately. Third, fixed effects were estimated in separate mixed models assessing the univariable associations between adherence to guidelines by chiropractors (dependent variable) and the following independent variables: gender, postgraduate education, country of practice, type of practice, years in practice since graduation, and familiarity with the clinical guidelines. The odds ratios (ORs), and 95% CIs were calculated and transformed into percentages by $=\frac{{e}^{\beta }}{1+{e}^{\beta }}$. Percentages were presented, as these are easier to interpret for clinicians. These percentages described the estimated percentages of subgroups of chiropractors (e.g., longer in practice) adhering to the guidelines. As the percentage of adherence to bed rest was so high, no uni- or multivariable generalized mixed model could be conducted due to limited discriminative ability. Finally, all independent variables were simultaneously entered as fixed effects in a multivariable mixed model. For ORs, predefined thresholds for weak (OR < 1.6), medium(1.6 < OR < 3.5), and strong (OR > 3.5) relations were defined a priori [30, 31]. All statistical analyses were performed in Statistical Package for Social Sciences for Windows (SPSS version 25).

## Results

### Response

**Fig. 1:**
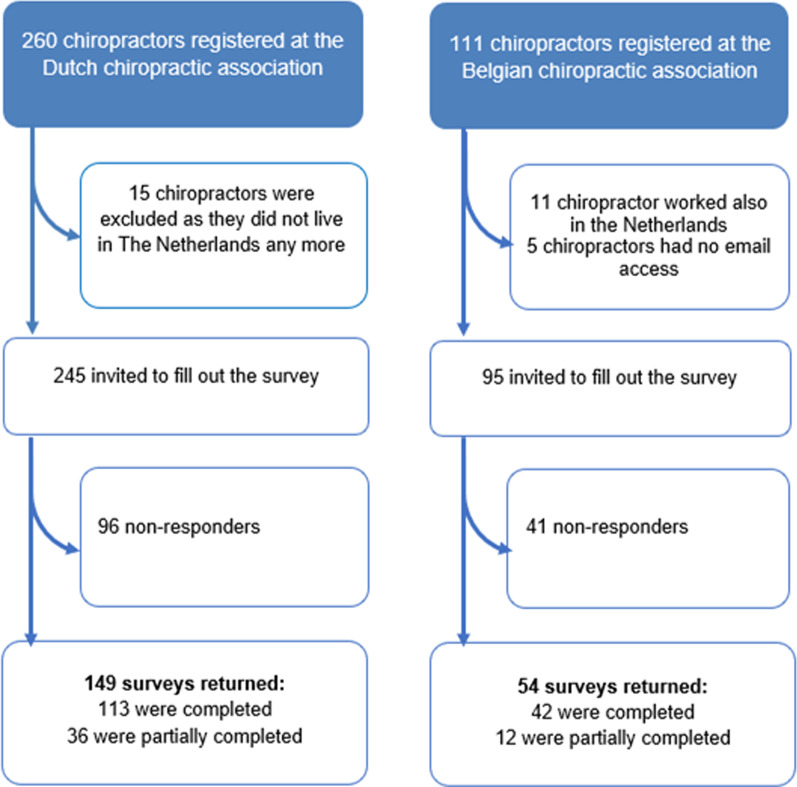
Survey response flow chart

Figure 1 indicates the flow of the recruitment and response. The data were collected in 2013. The overall response rate was 60% (n = 203/340), and was similar among the Dutch and the Belgian chiropractors. The majority (76%) of the respondents completed the survey. As the participants could not proceed to the next question before answering, but could stop at any time, the missing data were primarily from the last vignettes. Questions on vignette 1 were answered by 181 participants (89%), while the vignette 6 questions were completed by 159 participants (78%). Characteristics of the chiropractors were similar in both countries (Table 2), although Belgian chiropractors reported working more in solo practices as compared to the Dutch (63% vs 34%).

**Table 2:** Characteristics of responding chiropractors in the Netherlands and Belgium

	Chiropractors in the Netherlands (n = 149)	Chiropractors in Belgium (n = 54)
Gender (%)		
Female	40.3	27.8
Male	59.7	72.2
Age: mean (SD)	40.6 (11.4)	41.4 (14.1)
Nationality (%)		
Dutch	71.1	1.9
Belgium	5.4	90.7
Other	23.5	7.4
Years since qualification mean (SD)	14.2 (10.2)	16.0 (13.2)
Degree before chiropractic career (%)	39.6	31.5
Practice type (%)		
Solo practice	34.2	63.0
Group practice	49.0	29.6
Multidisciplinary setting	14.8	3.7
Other	2.0	3.7
X-ray facilities in the practice (%) (n = 144)		N/A*
X-ray machine	7.0	
Idexa scan	7.6	
None	85.4	
Postgraduate training (specialization) (%)		
Yes	41.6	27.8
No	58.4	72.2
Which specialization (% of all with postgrad/% of all respondents)		
Neurology	38.7/16.8	6.7/1.9
Sport	24.2/10.1	33.3/9.3
Paediatrics	19.4/8.1	26.7/7.4
Radiology	12.9/5.4	0
Clinical science	8.1/3.4	26.7/7.4
Other (e.g. dry needling, veterinary chiropractic)	33.9/14.1	33.3/9.3
Graduation from college (%)		
AECC	69.1	59.3
Other	30.9	40.7
Are you familiar with the practice guidelines?		
Yes	78.2	51.0
No	21.8	49.0

*SD* standard deviation

*Not applicable. Belgian chiropractors are not allowed to have x-ray facilities at their practice

### Practice guidelines

In total, 71% of the chiropractors reported being familiar with practice guidelines for the management of LBP patients. Most chiropractors familiar with the practice guidelines (80%) applied them in clinical practice. Dutch chiropractors [OR (CI): 3.2 (2.4;4.3)] and chiropractors who have post-graduate training [OR (CI): 1.9 (1.4; 2.6)] more often reported to be familiar with practice guidelines (medium strength association). Chiropractors longer than 20 years in practice reported less familiarity with practice guidelines [OR 0.6 (0.4; 0.8)] than those in practice shorter than 10 years (medium strength association). We didn’t find any associations between familiarity with guidelines and the other studied factors.

### Management

All treatments and care that chiropractors indicated they would provide for each vignette are detailed in Table 3. Chiropractors would employ a chiropractic adjustment (including SMT, Cox, Activator, Gonstead, and Thompson drop) in roughly 90% of the cases in all vignettes with the exception of vignettes 2 and 5, where the patient has already been treated before. For vignettes 2 and 5, a higher percentage of chiropractors chose no intervention as compared to the other vignettes. Psychological evaluation was not selected more than 20% in any of the vignettes. Noticeably, in the radicular symptoms’ vignettes (3 and 6) this is significantly lower than in the other vignettes. In vignettes 1, 3, 4, and 6, 30–50% of chiropractors reported using non-exercise modalities (e.g., heat, ice), which is markedly lower in the vignettes of patients who were already being treated (2 and 5) (resp. 15% and 17%). Chiropractors are more likely to give exercises to their patients in the chronic vignettes (4, 5 and 6 (resp. 49%, 54%, 31%)) than in the acute vignettes [1, 2 and 3 (resp. 30%, 35%, 25%)].

**Table 3:** Interventions undertaken or advice given by chiropractic respondents for the patient described in the vignette (%(n))

Response options	Vignette 1	Vignette 2	Vignette 3	Vignette 4	Vignette 5	Vignette 6
Treatment	n = 181	n = 176	n = 168	n = 163	n = 162	n = 159
No intervention expectant observant	4.4 (8)	22.7 (40)	3.6 (6)	3.7 (6)	13.6 (22)	3.8 (6)
Chiropractic adjustment (including SMT, Cox, Activator, Gonstead, Thomson drop)	92.8 (168)	59.1 (104)	91.1 (153)	92.6 (151)	72.8 (118)	89.9 (143)
Exercise	30.4 (55)	34.7 (61)	25 (42)	49.1 (80)	54.3 (88)	31.4 (50)
Education (back school)	26.5 (48)	26.7 (47)	29.2 (49)	36.2 (59)	40.1 (65)	28.9 (46)
Massage	28.2 (51)	15.9 (28)	19.6 (33)	27.6 (45)	19.1 (31)	16.4 (26)
Spinal traction (not flexion distraction)	6.1 (11)	10.8 (19)	16.7 (28)	6.7 (11)	5.6 (9)	16.4 (26)
Psychosocial evaluation by chiropractor	11.6 (21)	16.5 (29)	4.2 (7)	19 (31)	17.9 (29)	3.1 (5)
Non exercise modalities (eg. Heat, ice etc.)	53.6 (97)	15.3 (27)	41.1 (69)	30.1 (49)	17.3 (28)	29.9 (47)
Electrotherapy (eg. TENS, interferential, etc.)	0.6 (1)	1.1 (2)	1.2 (2)	0.6 (1)	0.6 (1)	0 (0)
Other treatment	18.3 (33)	22.2 (39)	19.0 (32)	16 (26)	14.2 (23)	14.5 (23)
How often do you treat the patient	n = 173	n = 116	n = 163	n = 151	n = 141	n = 152
Once a week	17.3 (30)	66.4 (77)	7.4 (12)	43.7 (66)	64.5 (91)	19.1 (29)
Twice a week	63.6 (110)	19 (22)	68.7 (112)	50.3 (76)	10.6 (15)	66.4 (101)
Three times a week	8.1 (14)	0 (0)	14.1 (23)	2 (3)	0.7 1)	9.9 (15)
Other	11 (19)	14.7 (17)	9.8(16)	4 (6)	24.1 (34)	4.6 (7)
Re-evaluation after … treatments; mean(SD)	3.6 (2.0)	3.2 (2.5)	3.8 (2.3)	4.5 (2.0)	3.6 (2.5)	4.1 (2.4)
*Advice on*						
Return to work	n = 179	n = 171	n = 167	n = 163	n = 162	n = 159
Return to normal work	3.4 (6)	3.5 (6)	1.2 (2)	38.7 (63)	29.6 (48)	10.7 (17)
Return to part time or light duties	38 (68)	49.1 (84)	23.4 (39)	51.5 (84)	54.9 (89)	54.7 (87)
Be off work until pain has improved	52.5 (94)	38 (65)	61.7 (103)	8.6 (14)	13 (21)	28.3 (45)
Be off work until pain has completely disappeared	4.5 (8)	5.8 (10)	8.4 (14)	1.2 (2)	2.5 (4)	4.4 (7)
Be off work for a further …. Weeks	1.7 (3) (mean 2.5 weeks, SD = 0.7)	3.5 (6) (mean 2.4 weeks, SD = 0.8)	5.4 (9) (mean 2.7 weeks, SD = 0.9)	0 (0)	0 (0)	1.9 (3) (2.2 weeks, SD = 0.8)
Bedrest	n = 179	n = 171	n = 167	n = 163	n = 162	n = 159
Avoid resting in bed entirely	6.7 (12)	9.9 (17)	5.4 (9)	33.1 (54)	29 (47)	10.7 (17)
Avoid resting in bed as much as possible	40.2 (72)	39.8 (68)	29.9 (50)	39.3 (64)	40.7 (66)	36.5 (58)
Rest in bed only when pain is severe	50.8 (91)	46.2 (79)	58.7 (98)	25.8 (42)	27.8 (45)	50.3 (80)
Rest in bed until pain improves substantially	2.2 (4)	4.1 (7)	6 (10)	1.8 (3)	2.5 (4)	1.3 (2)
Rest in bed until pain disappears	0 (0)	0 (0)	0 (0)	0 (0)	0 (0)	1.3 (2)
Stay active	n = 179	n = 171	n = 167	n = 163	n = 162	n = 159
Perform usual activities	1.7 (3)	2.3 (4)	0.6 (1)	23.9 (39)	21 (34)	3.8 (6)
Perform activities within the patient's tolerance	73.2 (131)	69 (118)	50.3 (84)	62.6 (102)	64.2 (104)	67.3 (107)
Perform only pain free activities	24 (43)	25.1 (43)	43.1 (72)	12.9 (21)	14.8 (24)	23.9 (38)
Limit all physical activities until pain disappears	1.1 (2)	3.5 (6)	6 (10)	0.6 (1)	0 (0)	5 (8)

Three-quarters of the chiropractors indicated treating the LBP patients one to two times a week, regardless of the duration of the complaint. Over 80% of the chiropractors referred patients more often in vignettes 2 and 5 (the vignettes where patients do not respond to treatment) compared to the other vignettes (less than 40%).

In the acute vignettes, chiropractors were led by the symptoms of the patients and advised in most instances to be off work until the pain had improved(51%). In the chronic vignettes (4, 5, and 6), the respondents most often indicated to advise the patients to take on light duties or normal work (80%). Chiropractors would advise more than 70% of the patients represented in the vignettes to stay active (within pain tolerance), except for vignette 2 of the acute patient who does not respond to treatment (51%).

More than 90% of the chiropractors advised their patients to avoid bed rest as much as possible, or only when pain is very severe.

### Adherence to clinical guidelines

The overall adherence to clinical guidelines for all six vignettes was 64.5% (CI 58.7–70.0). The chronic vignettes (73.4% (CI 66.7–79.2) were completed better than the acute vignettes (55.9% (CI 50.5–61.1) (Table 4). Results for the adherence to guidelines on the advice to return-to-work, bed rest and stay active can be found in Tables 5, 6 and 7. While advice to bed rest adherence was almost entirely according to the guidelines (98.5% (CI 97.3–99.1), advice to stay active and return-to-work were less adhered to [resp. 77.5% (CI 72.3–81.9); 59.4% (CI 55.2–63.4)]. Only for return-to-work, the overall adherence was scored better in the chronic vignettes [81.6% (CI 76.9–85.4)] than the acute [39% (CI 34.6–44.4)]. Dutch chiropractors were also more likely to act according to the guidelines on the advice to return-to-work [2.0 (1.3–2.9)] and advice to stay active [1.6 (0.8–3.1)] than the Belgian chiropractors (medium strength correlation).

**Table 4:** Practice guideline adherence for the management of low back pain by chiropractors in all vignettes: results of (univariable and multivariable) generalized mixed model

Univariable generalized mixed model	Practice guidelines adherence in the vignette (%(95% CI))	OR (95%CI)
Overall adherence for all six vignettes	64.5 (58.7–70.0)	
Overall adherence for the three vignettes describing patients with acute low back pain	55.9 (50.5–61.1)	
Overall adherence for the three vignettes describing patients with chronic low back pain	73.4 (66.7–79.2)	
**Postgraduate training**		
No (reference category)	64.6 (57.1–71.5)	
Yes	64.5 (54.9–73.0)	1.0 (0.6–1.7)
**Country where working**		
Belgium (reference category)	59.1 (47.5–69.8)	
The Netherlands	66.5 (59.8–72.7)	1.4 (0.8–2.4)
**Type of practice**		
Solo practice (reference category)	64.4 (55.3–72.5)	
Group practice	64.7 (56.9–71.8)	1.0 (0.6–1.7)
**Years in practice**		
0–10 years	67.4 (58.8–74,9)	
11–20 years	51.5 (40.5–62.4)	0.5 (0.3–0.9)
20+ years	73.6 (62.8–82.2)	1.3 (0,7–2.5)
**Familiar with guidelines**		
No (reference category)	59.3 (48.1–69.5)	
Yes	66.7 (59.6–72.9)	1.4 (0.8–2.4)

Multivariable generalized mixed models	Coefficient	OR	95% CI
Post-graduate education			
No (reference category)			
Yes	− 0.2	0.8	0.5–1.4
**Country where working**			
Belgium (reference category)			
Netherlands	0.4	1.5	0.8–2.7
**Type of practice**			
Solo practice (reference category)			
Group practice	− 0.1	0.9	0.5–1.5
**Years in practice**			
0–10 years (reference category)			
10–20 years	− 0.7	0.5	0.3–0.9
20+ years	0.4	1.4	0.8–2.7
**Familiar with guidelines**			
No (reference category)			
Yes	0.3	1.4	0.8–2.4

**Table 5:** Practice guideline adherence on advice to return to work by chiropractors in all vignettes: results of (univariable and multivariable) generalized mixed model

Univariable generalized mixed models	Practice guidelines adherence in the vignette (% (95% CI))	OR (95% CI)
Overall adherence for all six vignettes	59.4 (55.2–63.4)	
Overall adherence for the three vignettes describing patients with acute low back pain	39.4 (34.6–44.4)	
Overall adherence for the three vignettes describing patients with chronic low back pain	81.6 (76.9–85.4)	
**Postgraduate training**		
No (reference category)	56.5 (51.1–61.2)	
Yes	63.8(57.2–70.0)	1.4 (0.9–1.9)
**Country where working**		
Belgium (reference category)	47.6 (39.8–55.6)	
The Netherlands	63.6 (58.9–68.0)	1.9 (1.3–2.8)
**Type of practice**		
Solo practice (reference category)	58.6 (52.1–64.8)	
Group practice	60.0 (54.4–65.3)	1.1 (0.7–1.5)
**Years in practice**		
0–10 years	65.4(59.4–71.0)	
11–20 years	55.7 (47.9–63.3)	0.7 (0.4–1.0)
20+ years	52.5 (44.1–60.8)	0.6 (0.4–0.9)
**Familiar with guidelines**		
No (reference category)	54.2 (46.4–61.7)	
Yes	61.6 (56.6–66.3)	1.4 (0.9–2.0)

Multivariable generalized mixed models	Coefficient	OR	95% CI
**Post-graduate education**			
No (reference category)			
Yes	0.2	1.2	0.9–1.7
**Country where working**			
Belgium (reference category)			
Netherlands	0.7	2.0	1.3–2.9
**Type of practice**			
Solo practice (reference category)			
Group practice	− 0.2	0.8	0.6–1.2
**Years in practice**			
0–10 years (reference category)			
10–20 years	− 0.4	0.6	0.4–1.0
20+ years	− 0.5	0.6	0.4–0.9
**Familiar with guidelines**			
No (reference category)			
Yes	0.1	1.1	0.7–1.5

**Table 6:** Practice guideline adherence on advice on bedrest by chiropractors in all vignettes: results of univariable generalized mixed model

Univariable generalized mixed models	Practice guidelines adherence in the vignette [% (95% CI)]
Overall adherence for all six vignettes	98.5 (97.3–99.1)
Overall adherence for the three vignettes describing patients with acute low back pain	94.7 (92.3–96.3)
Overall adherence for the three vignettes describing patients with chronic low back pain	95.9 (93.7–97.3)

**Table 7:** Practice guideline adherence on the advice for staying active by chiropractors in all vignettes: results of (univariable and multivariable) generalized mixed model

Univariable generalized mixed models	Practice guidelines adherence in the vignette (%(95% CI))	OR (95% CI)
Overall adherence for all six vignettes	77.5 (72.3–81.9)	
Overall adherence for the three vignettes describing patients with acute low back pain	68.1 (61.8–73.8)	
Overall adherence for the three vignettes describing patients with chronic low back pain	83.8 (78.8–87.7)	
**Postgraduate training**		
No (reference category)	81.1 (75.1–86.0)	
Yes	71.0 (61.3–79.0)	0.6 (0.3–1.0)
**Country where working**		
Belgium (reference category)	72.3 (61.0–81.4)	
The Netherlands	79.3(73.4–84.1)	1.5(0,8–2.7)
**Type of practice**		
Solo practice (reference category)	79.0 (71.1–85.1)	
Group practice	76.3 (69.2–82.3)	0.9(0.5–1.5)
**Years in practice**		
0–10 years	77.6 (69.7–84.0)	
11–20 years	81.7(72.7–88.2)	1.3 (0.7–2.5)
20+ years	72.0 (60.2–81.3)	0.7 (0.4–1.5)
**Familiar with guidelines**		
No (reference category)	73.9 (63.5–82.2)	
Yes	79.0 (73.0–84.0)	1.3 (0.7–2.4)

Multivariable generalized mixed models	Coefficient	OR	95% CI
**Post-graduate education**			
No (reference category)			
Yes	− 0.6	0.5	0.3–1.0
**Country where working**			
Belgium (reference category)			
Netherlands	0.5	1.6	0.8–3.1
**Type of practice**			
Solo practice (reference category)			
Group practice	− 0.3	0.8	0.4–1.4
**Years in Practice**			
0–10 years (reference category)			
10–20 years	0.2	1.2	0.6–2.3
20+ years	− 0.3	0.8	0.4–1.5
**Familiar with guidelines**			
No (reference category)			
Yes	0.3	1.3	0.7–2.5

## Discussion

### Summary

Based upon the patient vignettes posed to the participating chiropractors, the management of LBP almost always includes chiropractic adjustment, which is consistent with the guidelines. Psychosocial evaluation is not commonly used and exercises are more often prescribed for patients with chronic LBP (45%) than for acute LBP (30%). The self-reported adherence to the guidelines in the six vignettes was at least two-thirds for management and advice. Our study is the first in Belgium and the Netherlands to examine guideline adherence among chiropractors for the management of LBP.

### Management

In other studies, the use of manipulation by chiropractors varies from 76 to 98% [17, 19, 20, 32]. One study [17] used similar vignettes to assess the management of LBP, resulting in a similar percentage of manipulation use (76%), but a lower percentage on advice to stay active (51%) than our acute vignettes (resp. 81% and 65%). Unfortunately, they only examined vignettes that described patients with acute LBP. However, they also included an extra vignette of a patient with suspicion of a vertebral fracture. Therefore, it is very hard to compare these percentages.

The percentage of chiropractors advising bed rest was found to be similar (resp. 6% and 8%) in two previous studies [17, 32] as in ours (3%). One study [19] found a similar percentage (74%) for advice to stay active among chiropractors in Norway (73%). It should be noted that one of the studies [32] was published 20 years ago. This may explain the differences in management, as in the past 20 years the emphasis on clinical guidelines, and advice to stay active and return-to-work has increased. Advice on return-to-work has not been investigated in other studies.

Most clinical guidelines recommend the use of the biopsychosocial model, especially for chronic LBP [33–35]. A low number of psychosocial evaluations were reported in our study. Why psychosocial evaluation by chiropractors is underutilized, whether this should and how this could be improved should be investigated more extensively.

Most chiropractors stated they would treat the patients described in the vignettes one to two times weekly, which was lower than in a systematic review that reported an average number of treatment sessions of 2–3 times per week [36]. However, advice on the optimal frequency of chiropractic treatment sessions is lacking. It should also be noted that most papers that were evaluated in this systematic review originated from countries where chiropractic is part of the public healthcare system and more well-known. Therefore, this may influence the frequency because of better insurance coverage or the support from the family physician who is more familiar with chiropractic.

### Adherence to the guidelines

Our results indicate that many other treatment modalities were used among chiropractors than adjustments, exercise, and advice to stay active and return-to-work. More than a quarter of the chiropractors indicated they would give the patient exercises (ranging from 25 to 54%), educate the patient about back pain (ranging from 26 to 40%), or advise non-exercise modalities (ranging from 15 to 54%). This led to many chiropractors ‘overtreating’ their patients when compared to the guidelines. This might be due to the fact that systematic reviews [37–40] do not demonstrate one treatment modality to be superior to others for LBP, but there are multiple modalities that are effective to a lesser degree. It is possible that the respondents choose multiple modalities with a lesser degree of effectiveness to increase the chances of a positive outcome. It is also possibly due to the Dutch and Belgian chiropractors not being up to date on the more recent literature.

Chiropractors from the Netherlands seem to be more adherent to the guidelines than the Belgian chiropractors with regard to management and advice to stay active and return-to-work, this is supported by the data that Belgian chiropractors stated that they were less familiar with the guidelines than the Dutch chiropractors, despite the availability of international and multidisciplinary guidelines. The fact that Belgium does not have any national chiropractic guidelines and the Netherlands does, should not lead to this difference as the Dutch guidelines were not published, but also do not differ from the published clinical practice guidelines. Despite the fact that the Belgian chiropractic profession recently made significant steps toward legislation, chiropractic is still seen as ‘alternative’ or ‘complementary’ in Belgium and the Netherlands [41, 42]. Improving guideline familiarity and adherence, as well as being part of a guideline development group for multidisciplinary guidelines or developing a national monodisciplinary guideline are likely to help the integration of chiropractic care into the public healthcare systems in Belgium and the Netherlands.

Previous studies [43, 44] demonstrate that chiropractors who had graduated more recently and chiropractors familiar with the guidelines adhere better to the guidelines. Our results suggest similar findings, but we cannot confirm their conclusions as our results showed only weak and not always consistent associations. The rationale is, younger chiropractors are more exposed to clinical practice guidelines during their education, while older chiropractors, educated before the introduction of guidelines, need more time before awareness and implementation of guidelines are realized.

### Strengths and limitations

This study provides an update on the management of LBP by chiropractors as well as providing data indicating chiropractors’ estimated self-reported adherence to clinical guidelines in Belgium and the Netherlands. There are a few important strengths and limitations to discuss. Firstly, there was a relatively high response rate (60%). While this is comparable to other surveys of chiropractic [19, 20], this might lead to response bias because non-responders may view management and clinical guidelines differently. Furthermore, it is possible that the views and opinions expressed by the participants are different than those in the broader chiropractic community in Belgium and the Netherlands because we only invited chiropractors that were members of their national organizations. However, they represent the majority of those in clinical practice; therefore, these results may be considered broadly generalizable.

Secondly, vignettes are cases used to obtain knowledge, attitudes, and/or opinions according to how the subjects would react in the hypothetical situation. Vignettes reduce courtesy bias and therefore may be more valid, meaning a chiropractor’s spontaneous reaction to a vignette may have a more valid outcome than if one was to pose direct questions to a chiropractor. In fact, vignettes may be a better reflection of what happens in ‘real life’ situations; and therefore, represent a more valid image of chiropractor’s opinion and/or actions in a certain situation [45] as it is known that practitioners’ behaviour may change when it is known that they are being observed. This is called the Hawthorne or observer effect [46], which is avoided by the use of vignettes. Furthermore, vignettes can be administered to large groups of subjects, contain easily adaptable variables, are cheap to administer, and reduce ethical concerns which may present during a consultation. Having said that, however, the most important limitation of this approach may be that participants give a socially desirable response and therefore, may not reflect their true feelings or opinions. Thirdly, most items of the survey and vignettes were adapted from previous surveys [17, 26]. While we tested the survey in a pilot, we did not examine test–retest reliability; therefore, we are not sure how consistent these results may be over time. More testing on the reliability and validity of these vignettes is advised for the future. Also, it has to be kept in mind that the classification of ‘in line’ or ‘not in line’ is open for interpretation, but based on the guidelines of 2013 and in consensus with multiple practicing chiropractors, who were also active in research.

Lastly, our data were collected in 2013; therefore, these results might not entirely be in-line with current thinking because of an influx of new graduates, the retirement of older chiropractors, and more focus in postgraduate education on adherence to practice guidelines. Multiple articles suggest that health care practitioners do not adequately follow guidelines for LBP, [47–49] while adhering to clinical practice guidelines should improve outcome [13, 49]. That outcomes have not improved in the last decades is supported by the Global Burden of Disease study, which examined self-reported LBP between the period 1990 to 2017. That study concluded that there was no improvement in the number of years lived with disability caused by LBP [50]. This would suggest that adherence to clinical practice guidelines, like other behaviour modifications in healthcare, is a slow process [49, 51] with the result that implementation of clinical guidelines has yet to be fully embraced. It is not likely that the chiropractic profession is different than other health care professions as no study has been published in the last decade, which would have drawn the awareness of chiropractors to the clinical practice guidelines. This seems an opportunity for the coming decade.

## Conclusion

Two-thirds of the chiropractors reported adhering to the guidelines for management and advice for LBP patients. The self-reported treatment modalities most frequently applied were chiropractic adjustments, supplemented by exercise and education. Although the adherence to the vignettes in this study is reasonably high, it could be improved further for management and advice on return-to-work. We found no strong associations between specific characteristics and self-reported adherence to guidelines. Practitioners should pay attention to the practice guidelines in acute LBP cases, especially when advising return-to-work.

## Supplementary Information

**Additional file 1.** Survey.

## Data Availability

The datasets generated and/or analyzed during the current study are available from the corresponding author on reasonable request.
